# *PPP2R2A* prostate cancer haploinsufficiency is associated with worse prognosis and a high vulnerability to B55α/PP2A reconstitution that triggers centrosome destabilization

**DOI:** 10.1038/s41389-019-0180-9

**Published:** 2019-12-10

**Authors:** Ziran Zhao, Alison Kurimchak, Anna S. Nikonova, Felicity Feiser, Jason S. Wasserman, Holly Fowle, Tinsa Varughese, Megan Connors, Katherine Johnson, Petr Makhov, Cecilia Lindskog, Vladimir M. Kolenko, Erica A. Golemis, James S. Duncan, Xavier Graña

**Affiliations:** 1 Fels Institute for Cancer Research and Molecular Biology, Temple University Lewis Katz School of Medicine, Philadelphia, PA 19140 USA; 2 Fox Chase Cancer Center, Philadelphia, PA 19111 USA; 3 Department of Immunology, Genetics and Pathology, Uppsala University, 752 36 Uppsala, Sweden

**Keywords:** Prostate cancer, Mitosis, Mitosis

## Abstract

The *PPP2R2A* gene encodes the B55α regulatory subunit of PP2A. Here, we report that *PPP2R2A* is hemizygously lost in ~42% of prostate adenocarcinomas, correlating with reduced expression, poorer prognosis, and an increased incidence of hemizygous loss (>75%) in metastatic disease. Of note, *PPP2R2A* homozygous loss is less common (5%) and not increased at later tumor stages. Reduced expression of B55α is also seen in prostate tumor tissue and cell lines. Consistent with the possibility that complete loss of *PPP2R2A* is detrimental in prostate tumors, *PPP2R2A* deletion in cells with reduced but present B55α reduces cell proliferation by slowing progression through the cell cycle. Remarkably, B55α-low cells also appear addicted to lower B55α expression, as even moderate increases in B55α expression are toxic. Reconstitution of B55α expression in prostate cancer (PCa) cell lines with low B55α expression reduces proliferation, inhibits transformation and blocks xenograft tumorigenicity. Mechanistically, we show B55α reconstitution reduces phosphorylation of proteins essential for centrosomal maintenance, and induces centrosome collapse and chromosome segregation failure; a first reported link between B55α/PP2A and the vertebrate centrosome. These effects are dependent on a prolonged metaphase/anaphase checkpoint and are lethal to PCa cells addicted to low levels of B55α. Thus, we propose the reduction in B55α levels associated with hemizygous loss is necessary for centrosomal integrity in PCa cells, leading to selective lethality of B55α reconstitution. Such a vulnerability could be targeted therapeutically in the large pool of patients with hemizygous *PPP2R2A* deletions, using pharmacologic approaches that enhance PP2A/B55α activity.

## Introduction

Protein phosphatase 2A (PP2A) exhibits tumor suppressor function^1^. Given that PP2A holoenzyme functions as a trimer, with activity and specificity modulated by myriad of positive regulatory subunits and inhibitory proteins, multiple potential mechanisms of tumor suppression have been proposed. The best-defined mechanisms include inactivating point mutations in the PP2A scaffold subunit in endometrial and other cancers, and the upregulation of PP2A inhibitors SET and CIP2A^2,3^.

The PP2A holoenzyme consists of a B regulatory subunit, associated with a core heterodimer composed of a catalytic (C) and a scaffold (A) subunit. There are four classes of regulatory subunits, including B/R2, B’/R5, B’’/R3, B’’’; these are of particular interest because they confer substrate specificity and regulatory, subcellular and cell type-dependent functionality^4,5^. Given multiple B regulatory subunits, and because the scaffold and catalytic subunits are each encoded by two genes, close to sixty distinct trimeric PP2A holoenzymes could assemble in cells^5^. There is growing evidence that inactivation of PP2A tumor suppressor activity could be mediated via alteration of B-regulatory subunits^2,5^. However, which of the many B regulatory subunits act as tumor suppressors is not well understood.

Several lines of data suggest that reduced expression of *PPP2R2A*, the gene encoding the B regulatory subunit B55α, promotes tumor pathogenesis. *PPP2R2A*, located at chromosome 8p21.2, is deleted at high frequencies in prostate, ovarian, and luminal type B breast cancers^6,7^. The *PPP2R2A* gene is also one of the most common breakpoints in prostate cancer (PCa)^8^. However, whether B55α is a genuine tumor suppressor in PCa is unknown, reflecting the lack of any rigorously defined mechanism of action of this protein in tumor suppression. As PCa is the most commonly diagnosed cancer in men in more developed countries, and the second most commonly diagnosed in men worldwide^9^, the high frequency of *PPP2R2A* alterations in PCa warrants its study.

Here, we first used public data from large cohorts of PCa patients to establish evidence for *PPP2R2A* as a haploinsufficient tumor suppressor. In evaluation of function, we found that reduced expression of B55α protein is common in PCa primary tumors and cell lines. Notably, even modest elevation of B55α expression inhibited proliferation, transformation and tumorigenesis specifically in PCa cells with reduced B55α expression. These phenotypes were based on B55α induction of defects in centrosomal structure and function, and represent the first defined link between B55α/PP2A and the vertebrate centrosome. Our data suggest that pharmacologic approaches stimulating B55-dependent PP2A activity in the large pool of patients with *PPP2R2A* hemizygous deletions should be explored as a potential novel therapeutic strategy in PCa patients.

## Results

### *PPP2R2A* is hemizygously deleted in PCa and its loss is associated with poorer prognosis

**Fig. 1:**
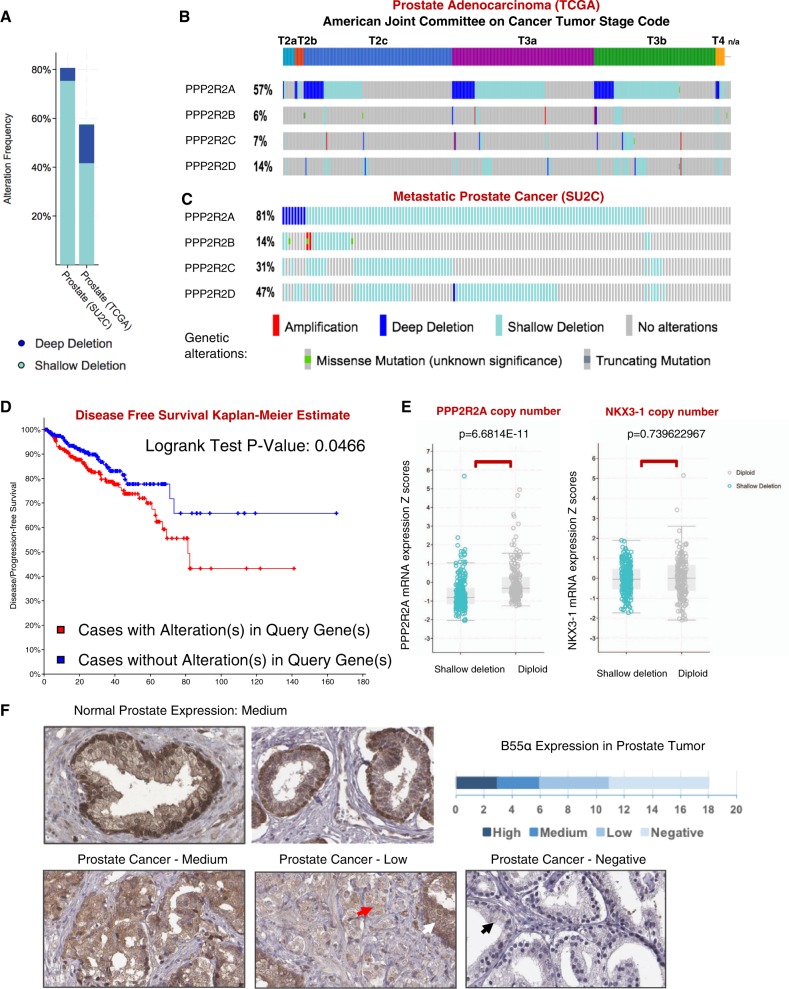
*PPP2R2A* DNA copy number and mRNA expression are reduced in prostate tumors and this correlates with worse tumor stage and poorer prognosis. **a** TCGA and SU2C database mining for DNA copy number in PCa. **b, c** Oncoprints show increased frequency of *PPP2R2A* hemizygous loss with higher AJCC tumor stage (TCGA data) and prostate cancer metastases (SU2C). See legend for genetic alterations. **d** Loss of *PPP2R2A* gene copy number correlates with poorer prognosis of PCa patients (*p* = 0.0466). **e** cBioportal analysis from TCGA PanCancer Atlas PCa data: *PPP2R2A* gene copy number alterations are associated with decreased mRNA expression in matched prostate tumors (*p* = 6.6814E−11, *t*-test). The same association is not seen with *NKX*3-1 (*p* = 0.739622967). **f** B55α expression is scored medium in normal prostate as compared to other tissues (see Suppl. Fig. 1G). B55α expression is low or negative in more than 60% of prostate tumors (Tissues were stained with anti-B55α (100C1) rabbit mAb).

Analysis of 492 prostate tumor genomes from the TCGA dataset (Fig. 1a) indicated that hemizygous loss of *PPP2R2A* occurred in ~42% (206/492) of prostate adenocarcinomas (shallow deletion). Frequency of hemizygous loss increased with tumor stage (Fig. 1b), and dramatically in metastatic tumors (SU2C dataset, >75%) (Fig. 1a, c). Importantly, hemizygous loss of *PPP2R2A* expression correlated with poorer prognosis, based on Kaplan-Meier estimates of disease-free survival (DFS) using TCGA data from patients with prostate adenocarcinoma (Fig. 1d, *p*-value 0.0466). This is consistent with the DFS calculated in an independent cohort with hemizygous loss of *PPP2R2A* (the MSKCC prostate adenocarcinoma data set, 194 tumors, *p*-value of 0.0053, Suppl. Fig. 1A-B). Homozygous loss (deep deletion) of *PPP2R2A* in prostate adenocarcinomas was less common (15%; TCGA), particularly in datasets reporting metastatic tumors (<5%; SU2C) (Fig. 1a). Surprisingly, homozygous loss shows a non-significant tendency to poorer prognosis (*p*-value 0.33, Suppl. Fig. 1A), indicating that there is no strong selection for loss of the second allele, conceivably because homozygous loss may be detrimental.

Comparable analysis of other orthologous B55 subunits from distinct chromosomal loci (*PPP2R2B*, *PPP2R2C*, or *PPP2R2D*) shows limited co-occurrence of their deletion with that of *PPP2R2A* in early stage (T2) tumors, but a striking increase in hemizygous deletion of *PPP2R2A* concurrent with loss of *PPP2R2B*, *PPP2R2C*, and/or *PPP2R2D* is observed in metastatic PCa (~60%) and to a lesser extent in T3 tumors (T3a: 19/156 = 12%; T3b: 36/132 = 27%) (Fig. 1b, c). Moreover, *PPP2CB*, which encodes the minor catalytic isoform of PP2AC, is often co-deleted with *PPP2R2A* as it is located on 8p21.2 (Suppl. Fig. 1C-D). In contrast, deletions affecting other PP2A B subunit families (R3 and R5) are infrequent (Suppl. Fig. 1C-D). Altogether, these data suggest that overall reduced B55/PP2A holoenzyme expression is selected during prostate carcinogenesis, but complete loss of B55α/PP2A is not.

Hemizygous loss of *PPP2R2A* was associated with reduced B55α mRNA expression (*p*-value 6.68 × 10^−11^), but not reduced expression of a known prostate tumor suppressor NKX3-1 (*p*-value 0.74), which is also located on Chr. 8p21.2 (Fig. 1e). Using immunohistochemical analysis of tissue microarrays, we evaluated B55α expression in the normal prostate versus prostate tumors (Fig. 1f, Suppl. Fig. 1E-G). In normal prostate, B55α staining is highest in the outer cuboidal cells in the prostate acini, lower in the inner luminal columnar epithelial cells and much lower in the fibromuscular stroma (Fig. 1f, upper panels). Based on intensity signal comparison, B55α expression is low or negative in 12 of 18 of prostate tumor cores (~67%) (Fig. 1f, lower panels), comparable to the frequency of observed hemizygous or homozygous loss. In tumor cores in which acinar structure is not completely disrupted adjacent to invasive tumor areas, highly reduced expression is more obvious in the tumor tissue (bottom middle panel). In summary, gene copy number loss and decreased RNA and protein expression support the notion that the gene encoding B55α could be a haploinsufficient tumor suppressor in PCa and that complete loss of B55α is detrimental in PCa and non-selected during tumorigenesis.

### Cell cycle defects in PCa cells with *PPP2R2A* deficiency

**Fig. 2:**
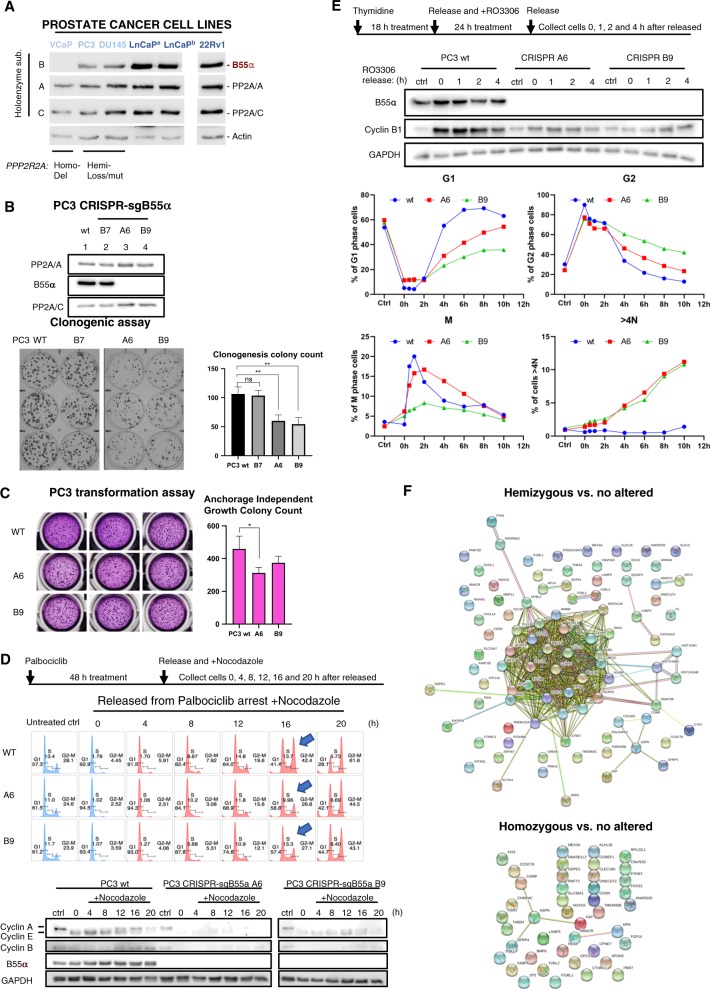
Complete loss of *PPP2R2A* reduces proliferation by slowing cell cycle progression. **a** B55α is not expressed in VCaP cells and is expressed at relatively low levels in PC3 and DU145 cells. Expression of B55α, PP2A/C and PP2A/A was determined by western blot. **b, c** Elimination of the remaining allele of *PPP2R2A* in PC3 cells via CRISPR knockout does not increase proliferation or transformation potential. **b** Western blot showing expression of B55α, PP2A/C and PP2A/A in PC3 wild-type, *PPP2R2A*-KO clones A6 and B9 and a control clone (B7) that did not exhibit deletion of *PPP2R2A*. Clonogenic assay and corresponding histogram showing colony count of these cell lines in triplicate. **c** Anchorage independent growth assay and corresponding histogram showing colony count of these cell lines in triplicate. Data represent mean ± SD. *p < 0.05; **p < 0.01; ns *p* > 0.05, *t*-test. **d** PC3 wild-type and *PPP2R2A*-KO clones A6 and B9 were arrested in G1 with palbociclib, released and allowed to progress to mitosis in the presence of nocodazole, collecting cells at times indicated in the timeline scheme. Cell cycle progression was analyzed by FACS/propidium iodide staining and the expression of indicated cyclins determined by immunoblot with juxtaposing images. **e** PC3 wild-type and *PPP2R2A*-KO clones A6 and B9 were synchronized via thymidine block followed by RO3306 treatment, released and collected at times indicated in the timeline scheme. Cell cycle progression was analyzed by FACS/phospho-Histone H3 (Ser10)/propidium iodide staining and cyclin B1 expression was determined by immunoblot. **f** Prostate Adenocarcinomas (TCGA Provisional) with hemizygous but not homozygous *PPP2R2A* alterations exhibit upregulation of many cell cycle genes as determined via STRING analysis. Experiments shown are representative of two independent experiments unless indicated.

To develop models for functional analysis, we assessed DU145, LNCaP, C4-2, and 22RV1 PCa cell lines for *PPP2R2A* expression and compared it to those with defined *PPP2R2A* genotypes, including normal BJ fibroblasts (wt), PC3 (shallow deletion/hemizygous loss), and VCaP cells (deep deletion/homozygous loss) (Fig. 2a, Suppl. Fig. 2A). B55α protein levels were reduced in PC3 cells and absent in VCaP cells, compared to the wild type controls. Interestingly, DU145 cells (with wt *PPP2R2A* copy number) also exhibited low B55α expression. Consistently, B55α protein expression in these cell lines corresponds with reported global mRNA analysis^10^ (Suppl. Fig. 2B).

We then used CRISPR to delete the remaining copy of *PPP2R2A* in PC3 cells. The resulting, PC3 B55α knockout cells formed significantly less and smaller colonies in clonogenic assays, and appeared less efficient in anchorage-independent growth (Fig. 2b, c). To determine the cause of the reduced proliferation, we synchronized PC3 wild-type and *PPP2R2A-KO* cells in G1 with a CDK4/6 inhibitor (palbociclib) or at the G2/M transition with a CDK1 inhibitor (RO-3306) and compared progression through the cell cycle upon release. Complete elimination of B55α resulted in cell cycle delays that were more prominent during S/G2 (Fig. 2d, note arrows) and G2/M phases and at mitotic exit and were accompanied by abnormal euploidy (Fig. 2e). Consistently, western blot analysis showed delays in the expression of G1/S and G2/M cyclins in cells released from the G1 and G2 arrests (Fig. 2d, e). Notably, in the TCGA dataset (Fig. 1a), tumors with hemizygous deletions of *PPP2R2A* exhibited cell cycle/mitotic signatures that included upregulation of multiple genes (33 genes from the 125 upregulated genes with log2 ratio >0.49 expression) (Fig. 2f). In contrast, there was no enrichment for cell cycle functions for the 45 genes that were upregulated in *PPP2R2A* homozygous deleted tumors (Fig. 2f, lower panel), indicating that *PPP2R2A* hemizygous tumors are more mitogenic. These data are in concordance with the observed lack of selection for loss on the second *PPP2R2A* allele with increased prostate tumor stage and metastasis (Fig. 1a–c).

### Reconstitution of B55α in PCa cells with low B55α expression inhibits growth and tumorigenesis by promoting mitotic arrest

To establish the mechanistic basis of *PPP2R2A* tumor suppressor activity in PCa, we first tried to develop stable overexpression cell lines for B55α in PC3 and DU145 cells. In spite of multiple attempts, only a single clone for each cell line with minimal expression of exogenous B55α (8%) was obtained (Suppl. Fig. 2C). In contrast, we had no difficulty in stably overexpressing B55α in rat chondrosarcoma^11^, human U2OS^12^, and 293 and 293T cells (Suppl. Fig. 2D). This suggested that increasing the low levels of B55α in these PCa cells was toxic, compatible with a proposed tumor suppressor activity and indicating a potential vulnerability in PCa cells with reduced B55α.

**Fig. 3:**
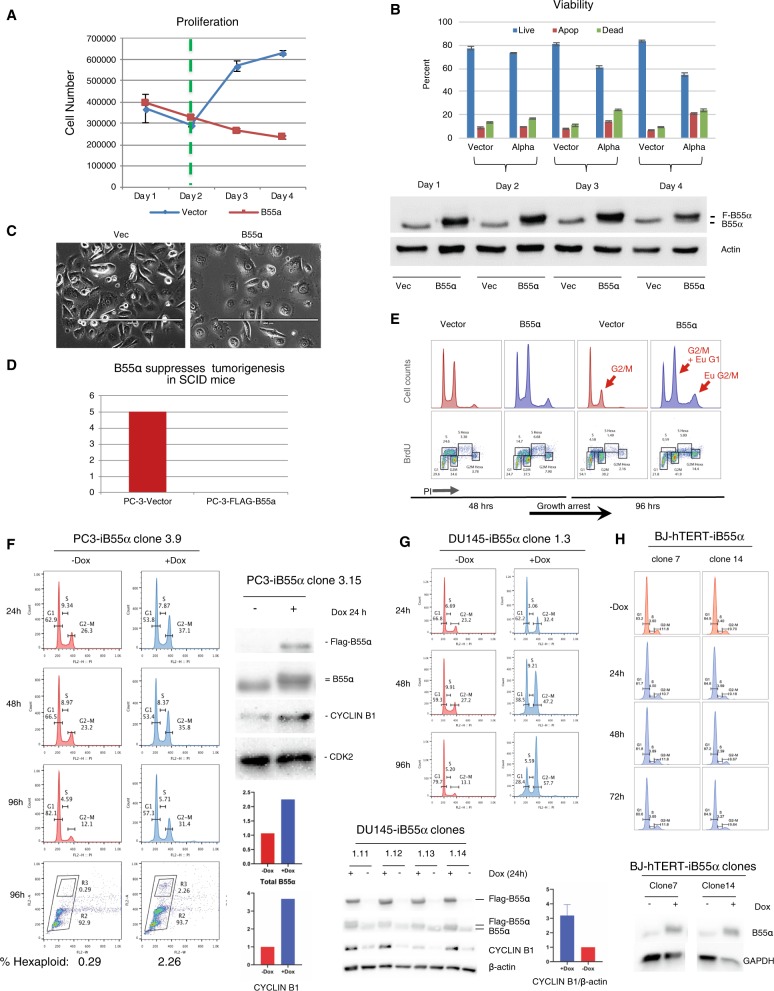
Reconstitution of B55α is toxic in PC3 and DU145 cells and inhibits transformation and tumorigenicity in SCID mice by inducing mitotic arrest. **a-d** Limited ectopic expression of B55α via lentiviral transduction **a** blocks proliferation (green dashed line shows end of Puromycin selection), **b** induces cell death and apoptosis, **c** results in a senescence-like flat cell morphology with large nuclei, and **d** suppresses tumor xenograft growth in SCID mice (mean tumor size 56 days post injection was 2117.37 +/− 462.14 mm^3^ [mean + /− SEM] in untreated cells and not detectable for Dox-treated cells). **e** Limited ectopic expression of B55α causes G2/M arrest and euploidy (PI/BrdU staining). PC3 cells are near triploid. G2/M = triploid G2/M cell population. Eu G1 = hexaploid G1 cells. Eu G2/M = hexaploid G2/M cells. **f–h** Inducible expression of B55α in PC3 and DU145 but not immortalized normal BJ-hTERT cells induces mitotic arrest. Dox inducible F-B55α PC-3 (**f**), DU145 (**g**), and BJ-hTERT cells (**h**) were generated by transduction with lentiviral Tet-on vectors and selected with zeocin or puromycin in tetracycline-free media. **f–h** Cells were induced with Dox and collected at the indicated times. DNA content was determined by PI/FACS analysis in at least two independent experiments. Expression of Flag-B55α, B55α, cyclin B1 and loading controls was determined via western blot analysis, quantitated and represented by histogram. Western blot analyses are representative of at least two independent experiments.

As an alternative approach, we transduced PC3 cells with lentiviruses directing the expression of Flag-B55α, or empty lentivirus controls, followed by rapid puromycin selection. The total level of B55α in these cells increased ~2-fold, mimicking B55α endogenous levels in cell lines without *PPP2R2A* deletions; we subsequently refer to this as a reconstitution model. PC3/Flag-B55α cells had reduced proliferation and viability, increased cell death and apoptosis, a flat morphology and increased cell and nuclear size (Fig. 3a–c, Suppl. Fig. 2E). Reconstitution of B55α also suppressed anchorage independent growth, and tumor xenograft growth in SCID mice (Suppl. Fig. 2F, Fig. 3D). Overall, these results were compatible with a specific intolerance for elevated B55α levels in PCa cells.

### B55α reconstitution is associated with mitotic defects

Suggesting a mechanistic cause for these phenotypes, PI/BrdU FACS analysis 96 h after transduction of Flag-B55α lentivirus showed striking accumulation of cells with G2/M DNA content, and the appearance of abnormal euploid cells (Fig. 3e). Using PC3, DU145, and LNCaP PCa cell lines, BJ fibroblasts and Tet-on lentiviral vectors, we generated multiple independent clones with inducible Flag-B55α. We were unable to perform comparable experiments in VCaP cells due to the slow growth and poor viability of this model. Doxycycline (Dox) induction of Flag-B55α approximately doubled the total level of B55α (reconstitution to wt levels), and consistently suppressed proliferation (Fig. 3f, g, Suppl. Fig. 3A–3B) and transformation (Suppl. Fig. 3A–3B) in both the PC3 and DU145 cell models. This was associated with accumulation of cells with G2/M DNA content (>30% for PC3, >55% for DU145) and ~3-fold accumulation of cyclin B1 in both models (Fig. 3f, g). Importantly, inducible expression of B55α in *PPP2R2A*-wt human BJ fibroblasts (~2.6 fold, Fig. 3h) and PCa LNCaP cells (1.9 fold, data not shown) did not result in accumulation of cells in G2/M, indicating that *PPP2R2A*-wt cells are not as vulnerable to increases in the expression of B55α.

**Fig. 4:**
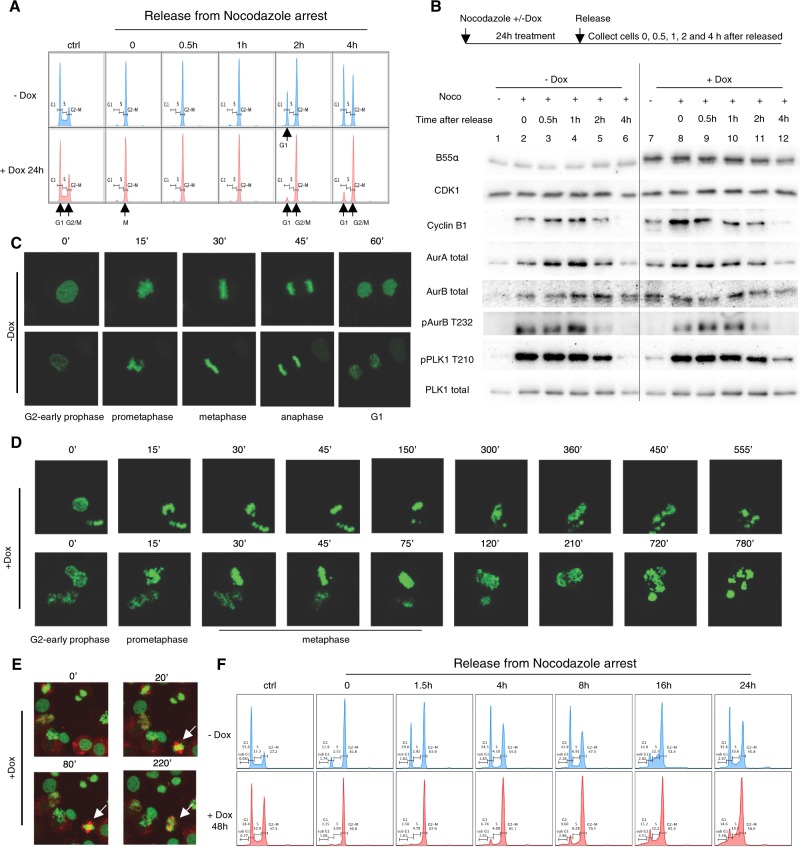
Reconstitution of B55α in PC3 and DU145 iB55α cells blocks mitotic exit as a result of extended mitotic checkpoint and chromosome segregation failure. **a, b** PC3 iB55α cells were synchronized by Nocodazole, shaken off, and reseeded as scheme shown. **a** B55α reconstitution slowed cell cycle progression through G2/M. **b** Expression of F-B55α, B55α, cyclin B1 and the indicated proteins was determined via western blot analysis. **c, d** Mitotic defects resulting from B55α reconstitution in DU145-iB55α-EGFP-H2B cells were determined by live imaging using confocal microscopy. Three fields of each treatment were imaged in at least three independent experiments. **c** Untreated cells went through normal mitosis in about 60 min. **d** Dox induced B55α caused an extended metaphase checkpoint followed by chromosome segregation failure and apoptosis. **e** Co-expression of EGFP-H2B and mRFP-α-tubulin in DU145-iB55α cells allowed visualization of bipolar spindles prior to centrosome collapse when B55α is reconstituted. Lens: ×20. **f** DU145-iB55α cells were synchronized by nocodazole as in **a**, reseeded and collected at the indicated time points. Cell cycle analysis was determined via PI/FACS in at least two independent experiments.

To refine the timing of action of B55α, PC3-iB55α cells were arrested with nocodazole +/− Dox, collected by mitotic shake-off, and allowed to resume mitosis. In the absence of reconstituted B55α, released cells progressed through mitosis and into G1, which resulted in cyclin B1 degradation, and decreased expression and/or phosphorylation of AURKA, AURKB, and PLK1. In contrast, cells with reconstituted B55α maintained a G2/M DNA content (Fig. 4a, b); surprisingly, these cells also degraded cyclin B1 and dephosphorylated AURKB and PLK1 after nocodazole release, albeit with slightly slower kinetics than in B55α-low cells, indicating activation of the anaphase promoting complex (APC), despite maintaining a G2/M DNA content (Fig. 4a, b). Of note, asynchronous control Dox-treated cells (without nocodazole treatment) accumulated AURKA and AURKB, PLK1 and P-PLK1-T210 (Fig. 4b, compare lanes 1 and 7), consistent with a fraction of the B55α-reconstituted cells accumulating in mitosis.

To determine the mitotic defect that prevented cells from reentering G1, we monitored chromosome dynamics in asynchronously growing DU145-iB55α-EGFP-H2B and PC3-iB55α-EGFP-H2B cells using time lapse microscopy. In the absence of Dox, essentially all cells progressed through mitosis in 60 min (Suppl. Movie 1, Fig. 4c). In contrast, Dox-treated cells in mitosis showed major defects in chromosome segregation that resulted in cell death (Suppl. Movie 2, Fig. 4d). These B55α high cells progressed to metaphase, but spent extended times, failing to fully arrange a narrow metaphase plate, and often exhibited misaligned chromosomes. The mitotic deficit did not reflect inability to form a spindle, based on analysis of Dox-induced DU145-iB55α-EGFP-H2B overexpressing RFP-α-tubulin (Fig. 4e). These cells initially formed normal-appearing bipolar mitotic spindles, but subsequently became disorganized as cells failed to progress beyond metaphase. Upon this extended checkpoint, defective chromosome segregation was observed, with chromosomes pulled in multiple directions for several hours, followed by cell death without undergoing cytokinesis. Based on FACS analysis, Dox-treated DU145-iB55α cells remained arrested with a G2/M DNA content for over 16 h and showed an increased sub-G1 fraction after 24 h (Fig. 4f). Similar chromosome segregation defects were observed using PC3-iB55α-EGFP-H2B cells (data not shown).

### Mitotic defects in B55α-reconstituted cells are linked to dephosphorylation of centrosomal proteins

**Fig. 5:**
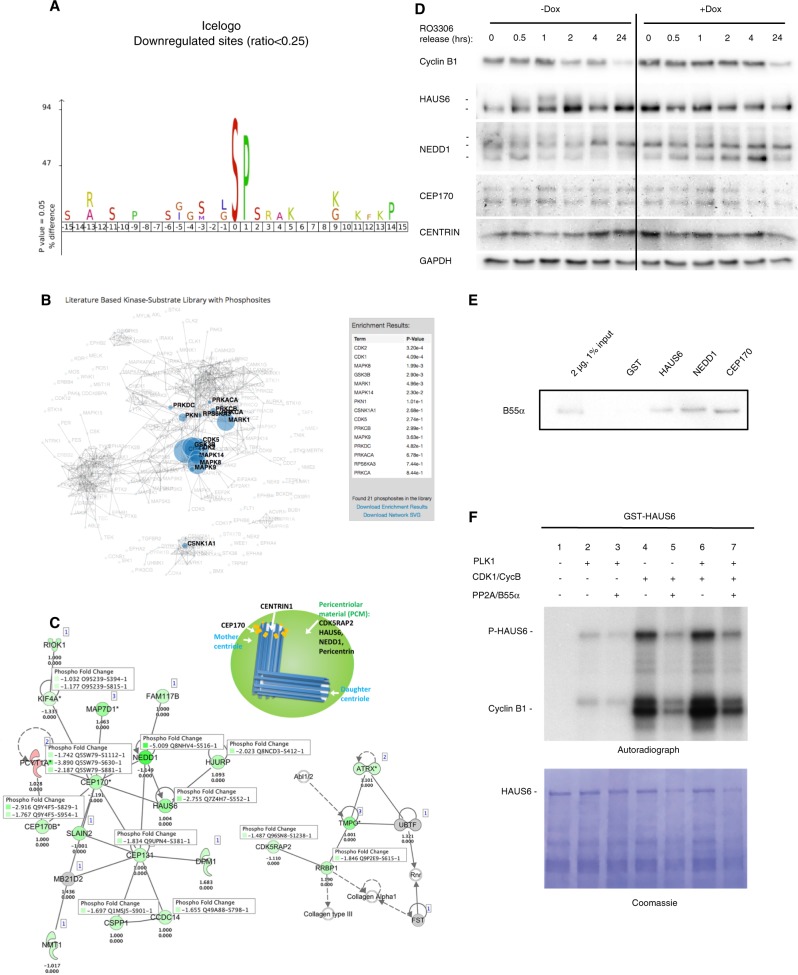
Mitotic defects in B55α-reconstituted cells are linked to dephosphorylation of centrosomal proteins. **a, b** SILAC**/**Phosphoproteome analysis was performed with PC3-iB55α cells treated with or without Dox. The consensus amino acid sequence of the top downregulated phosphopeptides was analyzed with (**a**) Icelogo and (**b**) the KEA2 algorithm, which predicts potential upstream kinases. **c** Ingenuity pathway analysis of phosphoproteome data shows that B55α reconstitution in PC3 cells reduces phosphorylation of multiple centriolar and PCM proteins. The cartoon displays the distribution of centriolar and PCM proteins. **d** Western blot analysis showed that B55α reconstitution in DU145 cells prevents phosphorylation of HAUS6 and NEDD1 in late G2/early mitosis (differently migrating protein species are indicated). **e** Purified GST, GST-HAUS6, GST-NEDD1, GST-CEP170 were incubated with 293T lysate and pull-downs analyzed by western blot for B55α. **f** Purified GST-HAUS6 was phosphorylated with PLK1 and/or CDK1/CYCLIN B in presence of γ-^32^P-ATP and dephosphorylated with purified trimeric B55α/PP2A complex (Suppl. Fig. 4B). Samples were resolved by SDS-PAGE, stained with Coomassie blue (lower panel) and analyzed by autoradiography (upper panel). Western blot and kinase/phosphatase analyses are representative of at least two independent experiments.

To gain insight into the observed mitotic defects, we used SILAC-based phosphoproteomics to compare PC3-iB55α cells +/−24 h of Dox treatment. Examination of global changes in the phosphoproteome showed clear enrichment for dephosphorylation of Ser/Thr-Pro motifs, representing potential CDK2/CDK1 sites^13^ (Fig. 5a, b), not seen in secondary upregulated phosphosites (Suppl. Fig. 4A). Ingenuity Pathway Analysis of these data identified 2 enriched protein networks containing dephosphorylated centrosomal proteins, as compared to cells not treated with Dox, including HAUS6, NEDD1, CEP170, and CDK5RAP2 (Fig. 5c). A number of these phosphoproteins localize to the centriole or the pericentriolar material (PCM) and have been implicated in centrosomal maintenance^14–17^. These signatures led us to focus on centrosomal defects associated with B55α reconstitution.

### B55α-reconstituted cells have centrosomal abnormalities that lead to centrosomal collapse after sustained checkpoint activation, causing cell death via apoptosis

We next explored changes in the expression and/or phosphorylation of pericentriolar material (PCM) proteins required for centrosomal integrity and γ-tubulin ring complex (γ-TuRC) nucleation (HAUS6, NEDD1). Consistently with the phosphoproteome data, reconstitution of B55α significantly delayed the appearance of slower migrating HAUS6 protein species (suggestive of differential phosphorylation) in cells progressing through mitosis (Fig. 5d). Similar results were observed for NEDD1, while no obvious migration changes were detected for CEP170 (Fig. 5d). GST-HAUS6, GST-NEDD1, and GST-CEP170 specifically interacted with B55α in pull-down assays from 293 cell lysates (Fig. 5e). Purified B55α/PP2A also efficiently dephosphorylated HAUS6 following their in vitro phosphorylation with CDK1 and PLK1 (Fig. 5f, Suppl. Fig. 4B). Taken together these data suggest that these proteins are B55α/PP2A substrates and that reconstitution of B55α in PCa cells with low B55α expression prevents their full phosphorylation potentially affecting centrosomal integrity.

**Fig. 6:**
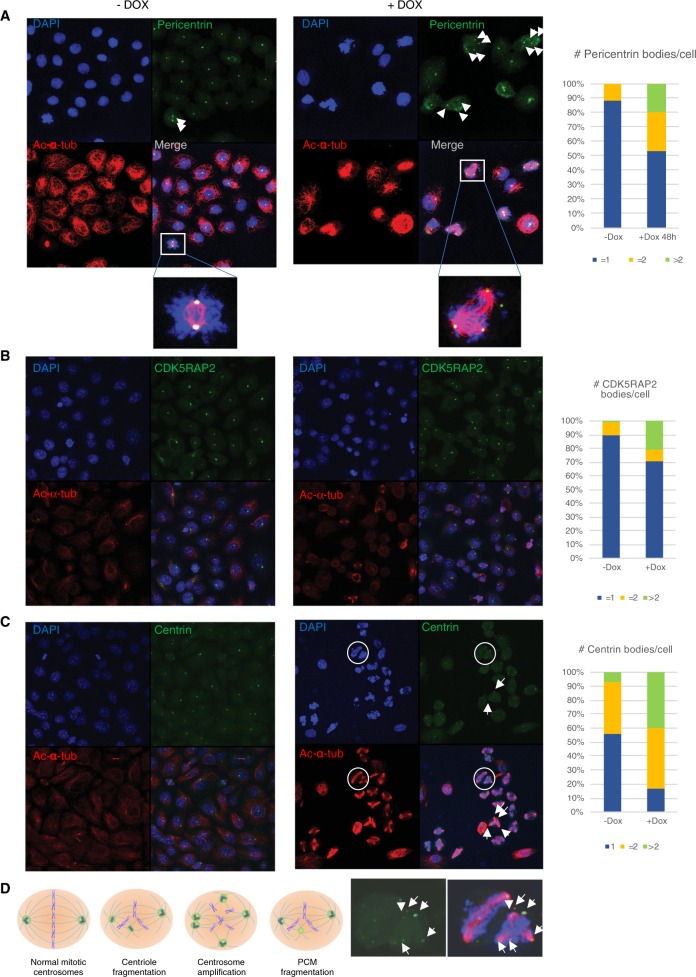
B55α reconstitution in DU145 cells promotes numerical centrosomal aberrations via PCM fragmentation and centriole overduplication. Immunofluorescent co-staining of (**a**) Pericentrin, (**b**) CDK5RAP2, or (**c**) Centrin1 with α-tubulin in control and Dox induced DU145-iB55α cells using specific antibodies. The number of (**a**) Pericentrin, (**b**) CDK5RAP2, and (**c**) Centrin1 bodies per cell were counted and expressed as bar graphs (on the right). Control cells show one Pericentrin, CDK5RAP2 or Centrin foci in interphase (G1/S/G2) (left panels). Dox-induced B55α expression results are consistent with centrosome overduplication, PCM fragmentation and disrupted spindles (right panels). DAPI was used to stain chromatin. Lens: ×63. **d** Diagram representing different mechanisms of centrosome disruption.

To further examine the relationship between B55α and centrosomal proteins, we performed immunofluorescence of DU145-iB55α cells with markers for the centriole (centrin) and the PCM (pericentrin, CDK5RAP2, NEDD1, HAUS6^18^). In Dox-untreated cells, pericentrin localized to one or two closely positioned centrosomes (the latter reflecting late S/G2 cells with completed centrosome duplication) (Fig. 6a), and mitotic cells showed normal spindles organized by two centrosomes in opposite poles (Fig. 6a). In contrast, Dox-induced DU145-iB55α cells accumulated in mitosis, and >20% of the cells exhibited more than two pericentrin foci, associated with multipolar spindles. This was similarly observed using CDK5RAP2 (Fig. 6b) and γ-tubulin to visualize centrosomes (Suppl. Fig. 5A). To determine if the centrioles are intact or become over-duplicated and/or fragmented, we co-stained for centrin and α-tubulin, which showed increased number of centrioles, in some cases with >4 foci; this suggested overduplication, although fragmentation cannot be excluded (Fig. 6c and d). These changes are accompanied by centrosomal collapse after extended metaphase, and occasionally also result in PCM fragmentation, as some foci contain PCM, but are centrin-negative (Fig. 6a, Suppl. Fig. 5B).

### Mitotic checkpoint activation contributes to centrosome collapse and cell death via apoptosis

**Fig. 7:**
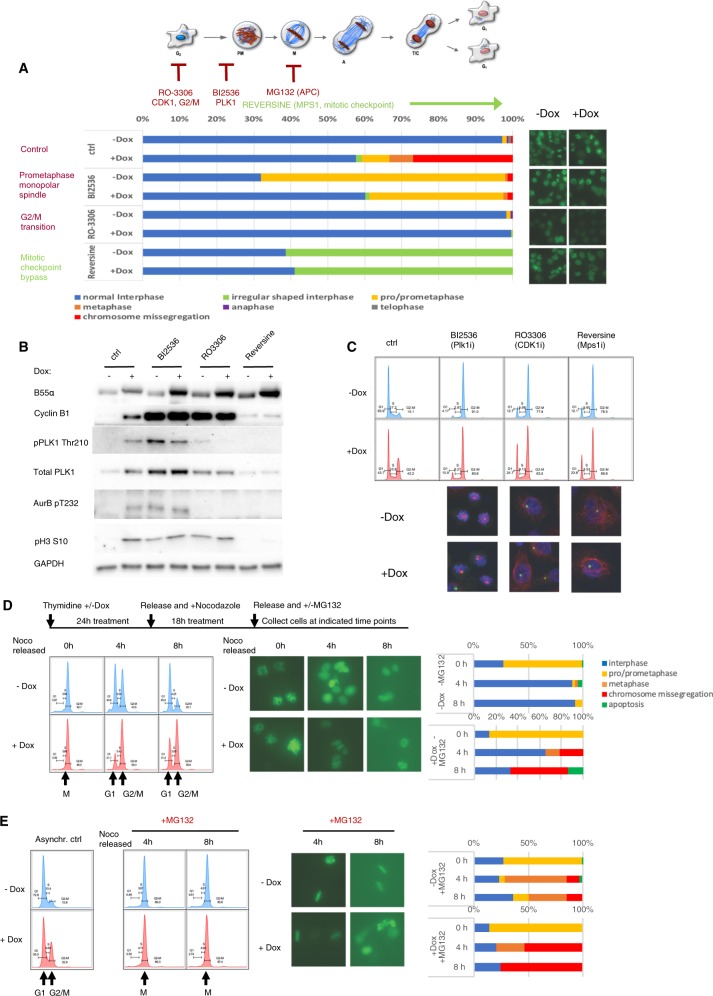
Pharmacological inhibition of the G2/M transition, centrosomal separation, cyclin B degradation or metaphase checkpoint activation in B55α reconstituted cells indicate that dephosphorylation of mitotic substrates leads to centrosomal amplification and/or weakening, causing its disruption following extended checkpoints. **a** (right panels) Images were taken after 20 h treatment of BI2536, RO-3306 or reversine, respectively, on thymidine synchronized DU145-iB55α-EGFP-H2B cells with or without Dox treatment, as the timeline scheme shown in upper left panel. Cells were counted and scored to the specified categories according to their chromatin state (Histone H2B-EGFP) (lower left panel) Lens: ×20. **b** Western blot analysis of cell lysates prepared from cells treated as in Fig. 7a. **c** DNA content was determined in cells treated as in Fig. 7a by PI/FACS analysis (upper panel) and immunofluorescence staining with DAPI, CDK5RAP2 and acetylated-α-tubulin (lower panel). **d, e** As shown in the timeline scheme, thymidine-nocodazole synchronized DU145-iB55α-EGFP-H2B cells treated (**d**) without or (**e**) with Dox were treated with 10 µM MG132 and images were taken at 0, 4, and 8 h respectively after treatment (middle panels). Lens: ×20. Chromatin states were scored via EGFP-H2B microscopic visualization, counted and represented as bar graphs (right panels). Cells collected at indicated times were also analyzed with PI/FACS (left panel).

Since chromosome segregation failure occurred following extended checkpoints, we asked if prolonged checkpoint activation was required for centrosome collapse in Dox-treated DU145-iB55α-EGFP-H2B cells. To this end we determined the effect of pharmacologic compounds that inhibit specific points during the G2/M transition and mitosis in chromatin/chromosome dynamics visualizing EGFP-H2B. Pharmacologic inhibition of CDK1 or PLK1 blocked the effect of doubling B55α expression, as cells released from a thymidine block incubated with the CDK1 inhibitor RO-3306 and Dox remained in G2 (large interphase nuclei), while cells incubated with the PLK1 inhibitor BI2536 and Dox remained in G2 or prometaphase (most with apparent monopolar spindles) (Fig. 7a, Suppl. Fig. 6A-B). The increased number of cells observed in G2 following treatment with BI2536 and Dox suggest that some of the Dox-treated cells were arrested in G2 or progressing slowly. To determine if mitotic checkpoint activation contributed to centrosome collapse, cells released from thymidine block were incubated with the MPS1 inhibitor reversine, which suppresses the spindle assembly checkpoint^19^, promoting passage to interphase. Interestingly, B55α-reconstituted cells treated with reversine also progressed to interphase although they did not undergo cytokinesis based on their DNA content (Fig. 7a–c, Suppl. Fig. 6C), suggesting that the extended checkpoint activation observed in Dox-treated cells in the absence of G2/M inhibitors promotes centrosome collapse. In controls, each inhibitor resulted in expected patterns of expression and phosphorylation for mitotic markers, DNA content and chromosomal/centrosomal structure (Fig. 7b, c, lower panels).

We next determined if preventing the metaphase to anaphase transition with the proteasome inhibitor MG132 in cells synchronized by double thymidine/nocodazole block could prevent defective chromosome segregation in Dox-treated DU145-iB55α-EGFP-H2B cells. In the absence of MG132, most nocodazole released cells without Dox treatment entered interphase by 4 h, while most Dox treated cells displayed chromosome segregation defects (Fig. 7d). As expected, DU145-iB55α-EGFP-H2B cells released from nocodazole arrest in the presence of MG132 without Dox arrested in metaphase by 4–8 h, with only a small fraction progressing to interphase or exhibiting chromosome segregation failure (Fig. 7e). In contrast, Dox-treated cells did not remain in metaphase, but failed to properly segregate chromosomes as the spindle collapsed (Fig. 7e). These data show that although DU145 cells expressing higher levels of B55α spend longer time in metaphase, extending this metaphase block further cannot prevent centrosome collapse (Suppl. Fig. 7A).

Finally, to determine the mechanism of cell death following failed chromosome segregation, Dox-treated cells released from thymidine block were incubated with the apoptotic inhibitor Z-VAD. Suppl. Fig. 7B shows that Z-VAD reduced apoptosis induced by B55α reconstitution to control levels. Interestingly, this allowed chromosome decondensation, premature cytokinesis and entry into G1 (Suppl. Fig. 7C). However, cytokinesis was defective, as lagging chromosomes were observed leading to micronuclei and aneuploidy (Suppl. Fig. 7C).

## Discussion

Previous studies have suggested a potential link of *PPP2R2A* alterations and PCa based on analysis of relatively small subsets of tumors^7,8^. Using TCGA and SU2C datasets of 492 and 150 PCa adenocarcinomas and metastasis, respectively, we for the first time show that hemizygous deletion of *PPP2R2A* is associated with poor tumor prognosis, and that the frequency of hemizygous deletion increases with tumor stage and is maximal in metastatic PCa. Based on mechanistic analysis, we propose the reduction in B55α levels associated with this hemizygous loss is necessary for centrosomal integrity and function in proliferating PCa cells, leading to selective lethality of B55α reconstitution in this form of cancer, and suggesting potential therapeutic vulnerabilities.

The selective hemizygous loss of *PPP2R2A* versus other B55-subunit encoding genes in PCa may reflect the fact that B55α is substantially more abundant than the other B55 family members in the prostate and other tissues;^20^ typically more than their combined expression^21^. The combined hemizygous loss of *PPP2R2A* with other subunit-encoding genes (*PPP2R2B-D*) could be explained either by hypothesizing these subunits each have distinct target specificity, or based on quantitative considerations, yielding further reductions in PP2A catalytic activity. Because of the abundance of *PPP2R2A*, homozygous loss of *PPP2R2A* will result in a greater reduction in B55/PP2A activity than the combined loss of *PPP2R2A* with *PPP2R2B-D*. We found that loss of the second allele of *PPP2R2A* is not selected for in aggressive PCa, and that a complete knockout of *PPP2R2A* in PC3 cells is deleterious, consistent with the slow growing properties of *PPP2R2A-null* VCaP cells. We also note that although VCaP cells are viable, they are the only *PPP2R2A*-null prostate cell line model we were able to identify in a broad survey of cell models, supporting the idea that complete loss of B55α function is deleterious.

Based on these data, we hypothesize co-occurrence of deletion of *PPP2R2B-D* alleles with hemizygous loss of *PPP2R2A* represents the largest reduction in B55/PP2A activity tolerated by PCa cells. A bioinformatic analysis of ovarian cancer tumors predicted that loss of *PPP2R2A* occurs early in ovarian tumor progression^22^. Although tumor suppressor activity of *PPP2R2A* has not been demonstrated in ovarian cancer, our data suggests that this may be likely. Interestingly, knockdown of *PPP2R2A* in MCF7 breast cancer cells induces proliferation^23^, supporting the possibility that B55α is tumor suppressive in breast cancer; further, *PPP2R2A* copy number loss is also observed in Luminal B breast cancer^6^. Hence, the results of this study are likely to be more generalizable to other cancers.

B55α/PP2A holoenzymes have been implicated in the dephosphorylation of signaling proteins such as AKT, and cell cycle regulatory proteins including retinoblastoma (RB) family proteins^4,24^. However, the most striking consequence of B55α reconstitution in PCa B55α-low cells are observed in G2/M. While our results do not rule out a role for B55α loss in contributing to prostate tumorigenesis by activating AKT and/or inactivating pRB, the gain and loss of function studies with B55α-low PCa cells described here demonstrate that maintaining reduced levels of B55α is critically important for mitotic survival and open a novel potential avenue for therapeutic intervention. Our data show that reconstitution of B55α exerts its major effect by leading to decreased phosphorylation of centrosomal proteins, centrosomal instability and collapse following an extended mitotic checkpoint and subsequently cell death via apoptosis (Suppl. Fig. 7A).

Importantly, vertebrate B55α has not previously been implicated in the control of centrosomal biology. We show here that B55α regulates the phosphorylation state of multiple proteins critical for centrosomal integrity (HAUS6, NEDD1, CEP170). Most of the phosphorylation sites enriched by reconstitution of B55α are proline-directed Ser sites (Fig. 5a). This is consistent with a possible B55α consensus dephosphorylation site for mitotic substrates^25^. HAUS6 is a subunit of the 8-subunit Human Augmin Complex localized to microtubules in mitosis. Disruption of any of its subunits results in destabilization of kinetochore microtubules, associated with centrosome fragmentation and the accumulation of multipolar spindles^14^. Of note, HAUS depletion appears to generate spindle force imbalance that centrosomes cannot sustain^14^. This is consistent with our finding that an extended mitotic checkpoint is required for centrosome collapse when B55α expression is increased, as reversine bypasses both the mitotic checkpoint and centrosomal collapse promoting entry into G1. Among the other B55α-influenced proteins, NEDD1 targets **γ**TuRCs to the centrosome^16^ and NEDD1 phosphorylation regulates spindle assembly^15^. Our data are consistent with B55α/PP2A controlling dephosphorylation of HAUS6 and NEDD1 in a manner that is important for centrosomal integrity. The reduced phosphorylation of several centriolar and PCM proteins key for various steps in centrosomal maturation upon B55α reconstitution may cooperate to promote centrosomal instability and chromosome segregation failure in cells addicted to low levels of B55α.

The high sensitivity to increased B55α expression in PC3 and DU145 cells, but not other cancer cell lines and normal BJ-fibroblasts, exposes a susceptibility that could potentially be targeted pharmacologically by PP2A activating drugs with preference for B55 family PP2A holoenzymes. Of note, drugs that upregulate PP2A activity and kill cancer cells have been described, including two SMall Activators of PP2A (SMAPs), FTY720, and OP499^26–29^. The discovery of such compounds suggests that identifying or generating more selective activators of PP2A through medicinal chemistry is feasible^26,27^. This makes B55/PP2A holoenzymes highly attractive for drug development and *PPP2R2A* hemizygous deletion would be an excellent biomarker to determine the subset of patients with drug sensitivity.

## Materials and methods

### Cell culture and cell lines

All cell lines were obtained from ATCC and cultured in DMEM/10% Tet-free FBS as described previously^12^ and tested for mycoplasma annually. For stable expression of B55α, PC3 and DU145 cells were transfected with pMSCV-puro-Myc-B55α, while 293 and 293T cells were transfected with pCPP-Flag-B55α, followed by puromycin selection. For transient lentivirus generation, PC3 cells were transduced with pCPP-Flag-B55α or pCPP lentiviruses as described previously^11^. For stable Dox-inducible Flag-B55α clone generation, PC3 and DU145 cells were transduced with FU-tetO-Flag-B55α and FUdeltaGW-rtTA lentiviruses, while BJ-hTERT fibroblasts and LNCaP cells were transduced with pCW57.1-Flag-B55α lentiviruses. DU145 iB55α clones were transfected with pBOS-EGFP-H2B to generate DU145-iB55α-EGFP-H2B cells and subsequently with pmRFP-α-tubulin_C1. To delete B55α, PC3 cells were transduced with lentiCRISPRv2-sgB55α and selected with puromycin.

### Plasmids

Listed in Suppl. Table 1.

### Cell cycle, proliferation and transformation analysis and kinase assays

For G1 arrest, cells were treated with 1 µM palbociclib for 48 h. Mitotic shake-off cells enriched via 10 nM nocodazole treatment were either reseeded for mitotic arrest and release or treated with 10 µM MG132 for metaphase arrest. For G2/M arrest or bypass, cells synchronized with 2 mM thymidine +/− Dox were treated with 10 µM RO3306, 0.1 µM BI2536, or 5 µM reversine. Z-VAD-FMK (50 µM) was used to inhibit apoptosis.

Substrates for phosphatase assays were generated by phosphorylating GST-fusion proteins as described^12^, followed by 1 h incubation with Flag-B55α/PP2A complexes purified from 293T-Flag-B55α clones (Suppl. Fig. 2D). Samples were resolved by SDS-PAGE and substrate dephosphorylation determined by autoradiography.

DNA content and BrdU-PI cell cycle analyses were performed as described previously^30^. Cell proliferation and viability was determined using the ViaCount method (Millipore 4000-0040). For clonogenic assays, cells were cultured for 14 days and stained with crystal violet. Anchorage independent assays were performed in 24-well plate as previously described^31^.

### Assessment of in vivo tumor growth

Cells (1 × 10^6^) were injected s.c. in the flanks of 6-week-old male C.B17/Icr-scid randomly assigned mice as described previously^32^. None were excluded from the analysis. Tumors were measured and volumes calculated [volume = 0.52 × (width)^2^ × length](no blinding was used).

### Immunoblots, tissue microarrays, Immunohistochemistry and Immunofluorescence

Western blot analysis was performed as previously described^33^ using antibodies detailed in Suppl. Table 2. Tissue microarrays (TMAs) were developed from donor blocks acquired from the archives at the Department of Pathology of Uppsala University Hospital, under an approved protocol (Ups 02–577), and included 6 normal prostate and 18 PCa samples; these were stained with anti-B55α antibodies and analyzed as previously described^34^. Immunofluorescence was performed as described previously^35^. For live imaging, cells were seeded in 8-well glass bottom µ-slides (iBidi #80827) and maintained with microscope stage TOKAI HIT GSI2 incubation system. Three positions of each sample were tracked for 24–48 h.

### Phosphoproteomic mass spec analysis

For SILAC labeling, PC3, PC3-iB55α, or DU145-iB55α cells were adapted as described^36^ and treated with 2 µg/ml Dox for 48 h as indicated. To identify potential substrates and downstream targets of B55α in +/−Dox treated PC3-iB55α cells, global phosphoproteomics analysis was performed as previously described^36^. Protein identification was performed by searching MS/MS data against the Swiss-prot human protein database using andromeda 1.5.6.0 built in MaxQuant 1.6.1.0^36–38^.

### Bioinformatics and biostatistics analysis

Gene copy number and Kaplan-Meier survival analyses were obtained using TCGA and SU2C datasets using MSKCC Bioportal tools^39–42^. *P*-values for TCGA, MSKCC, and SU2C datasets were determined by logrank or student’s *t*-test. Data for *PPP2R2A* mRNA expression in cultured prostate cells were downloaded from NCBI's Gene Expression Omnibus (GEO accession number GSE19426)^10,43,44^. The Icelogo and Kinase Enrichment Analysis 2 (KEA2) applications^13,45^ were used to determine a consensus dephosphorylation site and to predict kinases that target these sites in induced PC3-iB55α cells (Suppl. Table 3). Ingenuity pathway analysis (IPA, Qiagen) was performed to identify networks, using compiled expression ratios from the proteome and phosphoproteome of PC3-iB55α cells +/−Dox (Suppl. Table 4). STRING analysis was used to determine gene expression enrichment association with *PPP2R2A* status using PCa TCGA Provisional gene expression enrichments (Suppl. Table 5)^46^. Values represent the mean +/− standard deviation (SD) of triplicates.

## Supplementary information

Supplementary Figure and table legends

Supplementary figures

Supplementary Movie 1

Supplementary Movie 2

Supplemental Table 1

Supplemental Table 2

Supplemental Table 3

Supplemental Table 4

Supplemental Table 5
