# Supplementary material for: PPP2R2A prostate cancer haploinsufficiency is associated with worse prognosis and a high vulnerability to B55α/PP2A reconstitution that triggers centrosome destabilization
Source: Oncogenesis. 2019 Dec 10;8(12):72. doi: 10.1038/s41389-019-0180-9 (PMC6904742; doi:10.1038/s41389-019-0180-9)
Supplement: Supplementary file 1 — Supplementary Figure and table legends [file 41389_2019_180_MOESM1_ESM.docx]

**Supplementary Figures**

**Supplemental Fig. 1.** *PPP2R2A* is frequently hemizygously deleted in prostate cancer and the hemizygous loss correlates with poorer prognosis. **A, B** Disease-free survival of *PPP2R2A* homozygous or hemizygous deletions versus no *PPP2R2A* alteration prostate cancer tumors from **A** TCGA and **B** MSKCC studies. **C,D** Oncoprints show increased frequency of *PPP2R2A* hemizygous loss with **C** higher tumor stage (TCGA dataset) and **D** prostate cancer metastases (SU2C dataset). **D** Increased frequency of hemizygous loss of other B55 genes is associated with metastatic PCa. **E,F** Rabbit monoclonal antibodies (100C1) specifically recognize B55α and do not cross-react with other B55 subunits as determined by **E** immunofluorescence and **F** western blot analysis of PC3 cells and PC3 B55α CRISPR knockout cells. B9, B2, A6 and A4 are independent CRISPR knockout clones. A4 expresses a truncated B55α at lower levels indicated as *. **G** B55α expression in several tissues was determined using rabbit monoclonal antibodies (100C1). Expression intensity was scored (negative, low, medium or high).

**Supplemental Fig. 2.** Reconstitution of B55α is toxic in PC3 cell lines, but has no effect in 293 and 293T cells. **A** Quantitation of B55α levels in two separate experiments as shown in **Fig. 2A**. Detection of signal in VCaP cells is due to nonspecific background (mean expression is shown). **B** *PPP2R2A* mRNA expression in 2D and 3D cultures of prostate cells (NCBI’s Gene Expression Omnibus GSE19426) (10). **C** Only one clone of PC3 and DU145 (not shown) survived selection and expression was only 8% over endogenous B55α expression (left panel). Histogram showing the quantification of western blot (right panel). **D** Multiple clones of 293 and 293T cells stably expressing Flag-B55α at levels higher than endogenous B55α expression levels in the parental cell lines. **E** Limited ectopic expression of B55α using lentiviral transduction results in large nuclei. Images of EGFP-H2B labeled nuclei (upper panels) and corresponding bright field (lower panels) are shown. **F** Soft agar growth assays showed that PC3 cells ectopically expressing B55α fail to grow in an anchorage independent manner.

**Supplemental Fig. 3.** Induction of exogenous B55α expression in **A** PC3 and **B** DU145 cells inhibits proliferation in clonogenic assays (left panels) and anchorage independent growth in soft agar (right panels).

**Supplemental Fig. 4. A** Global upregulated phosphosites were determined by SILAC-based phosphoproteomics of PC3-iB55α cells +/- 24 hours of Dox treatment. The amino acid sequence of the top upregulated phosphopeptides was analyzed with Icelogo (right panel) and the KEA2 algorithm (left panel), which predicts potential upstream kinases. **B** Affinity purified PP2A/B55⍺ holoenzymes stained with Coomassie Blue. Briefly, 293T cells stably expressing Flag-B55α are lysed as previously described [33] and incubated with Flag agarose beads. Following capture, Flag-B55α containing PP2A holoenzymes are eluted from beads using excess Flag peptide and assessed for expression levels and purity by western blot and Coomassie Blue staining following SDS page.

**Supplemental Fig. 5.** Localization of centrosomal proteins within B55α low and high cells. **A** γ-tub/α-tubulin and **B** NEDD1**/**centrin were detected via immunofluorescence staining in DU145 iB55α control or Dox treated cells.

**Supplemental Fig. 6.** Pharmacological inhibition of the G2/M transition, centrosomal separation, or metaphase checkpoint activation in B55α reconstituted cells indicate that dephosphorylation of mitotic substrates leads to centrosomal amplification and weakening, causing its disruption following extended checkpoints. DU145 iB55α cells were treated with **A** BI2536, **B** RO-3306, or **C** reversine with (right) or without (left) induction of exogenous B55α, followed by IF staining for CDK5RAP2/α-tubulin.

**Supplemental Fig. 7. A** Schematic representation of results in Fig. 7 and Suppl. Figs. 6 and 7. **B** Histogram of viability assay demonstrating Dox-treated DU145 iB55α cell death via apoptosis. **C** Pan-apoptosis inhibitor ZVAD treatment rescued B55α reconstituted cells from apoptotic cell death and promoted defective cytokines and G1 entry. Live imaging frames showing the same cell at the indicated time points. Arrows indicate micronuclei. Lens: 20x.

**Supplemental movies**

Movies for Figure 4

**Supplemental Tables**

**Supplemental Table 1:** Plasmids

**Supplemental Table 2:** Antibodies

**Supplemental Table 3:** Phosphopeptide data used for Icelogo and KE2A

**Supplemental Table 4:** Kinome Proteome Phosphoproteome values for IPA

**Supplemental Table 5:** PCa TCGA Provisional gene expression enrichments
