# Supplementary material for: PPP2R2A prostate cancer haploinsufficiency is associated with worse prognosis and a high vulnerability to B55α/PP2A reconstitution that triggers centrosome destabilization
Source: Oncogenesis. 2019 Dec 10;8(12):72. doi: 10.1038/s41389-019-0180-9 (PMC6904742; doi:10.1038/s41389-019-0180-9)
Supplement: Supplementary file 2 — Supplementary figures [file 41389_2019_180_MOESM2_ESM.pdf]

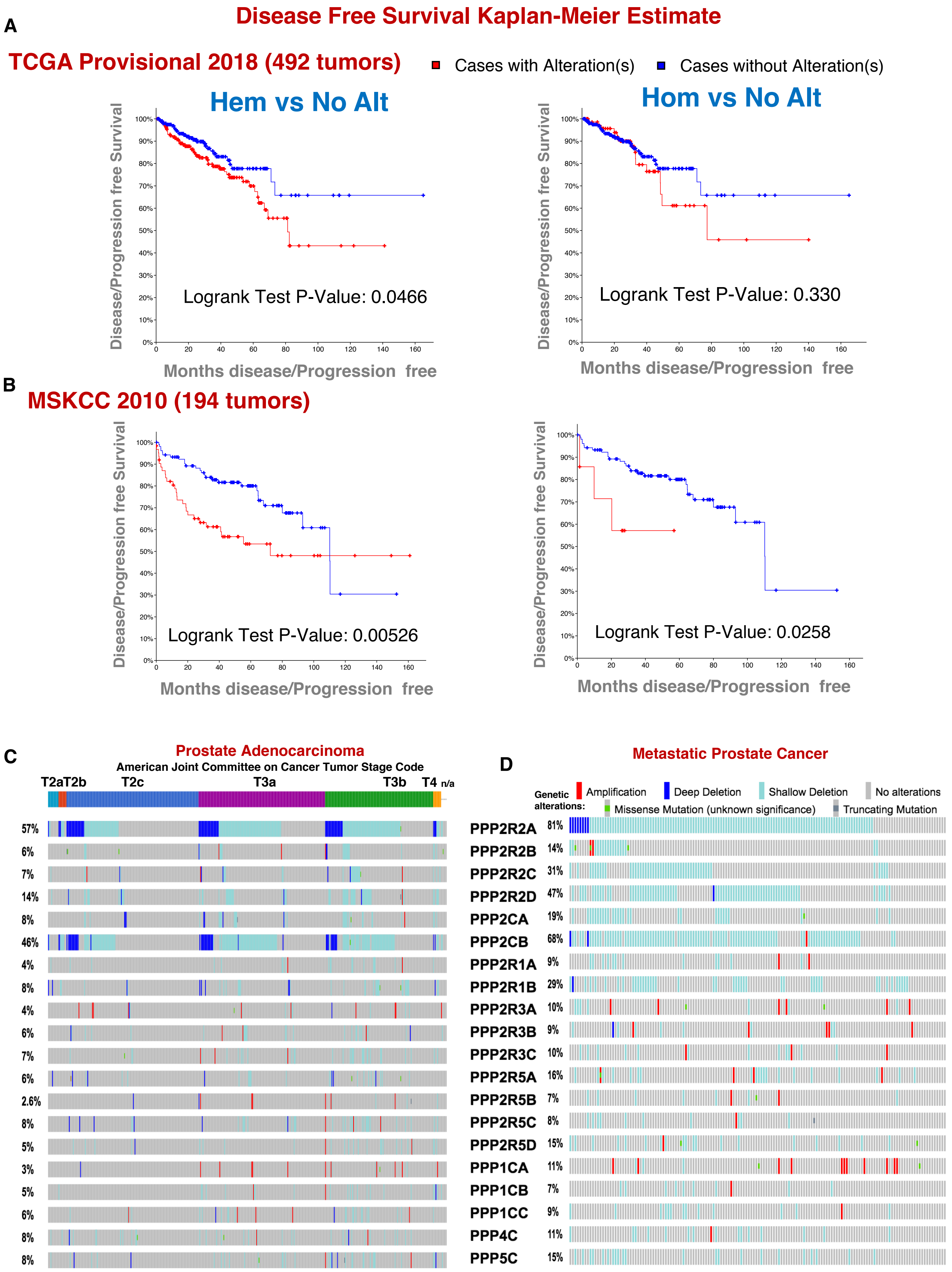

Supplemental Fig. 1.

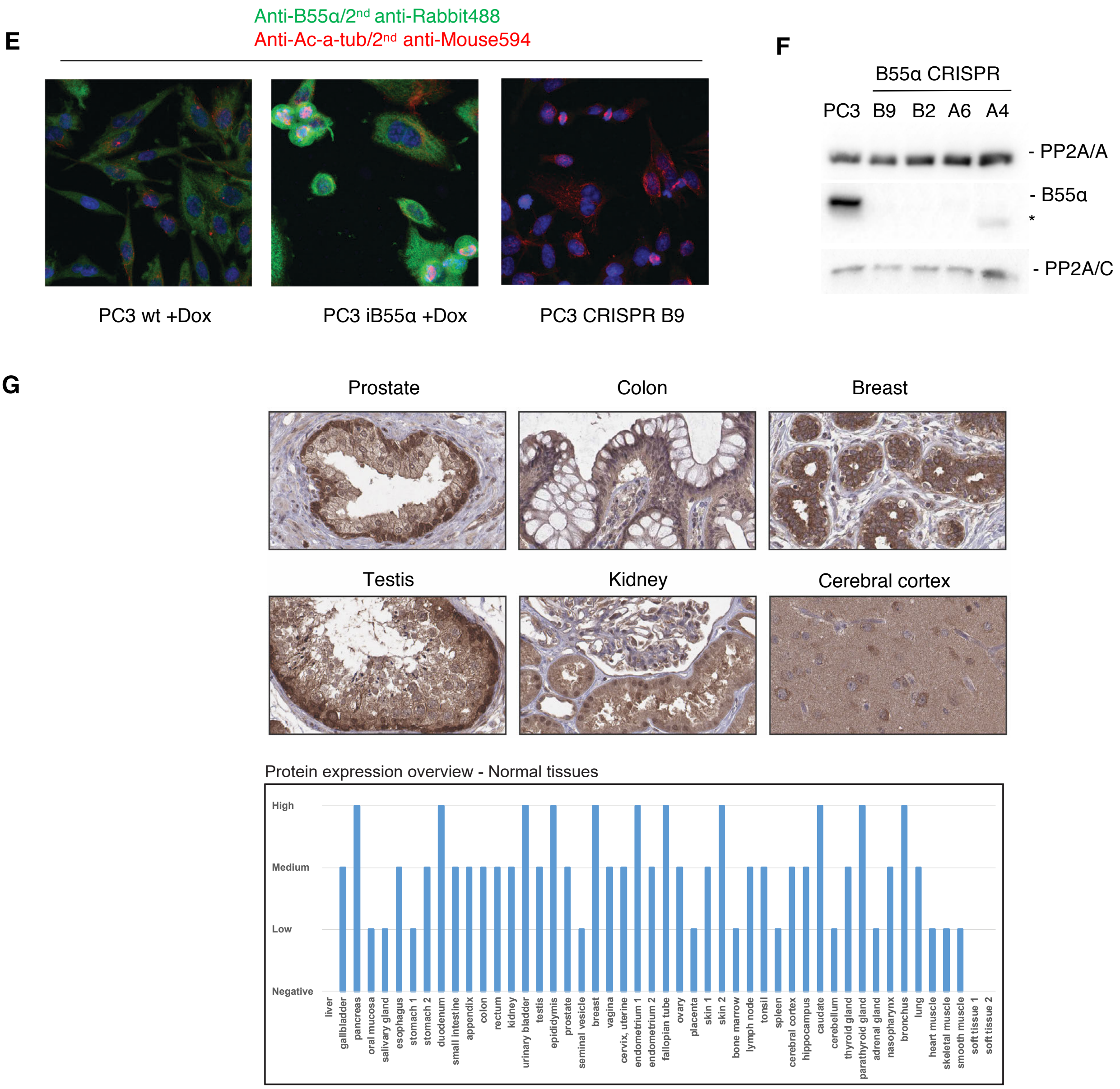

Supplemental Fig. 1.

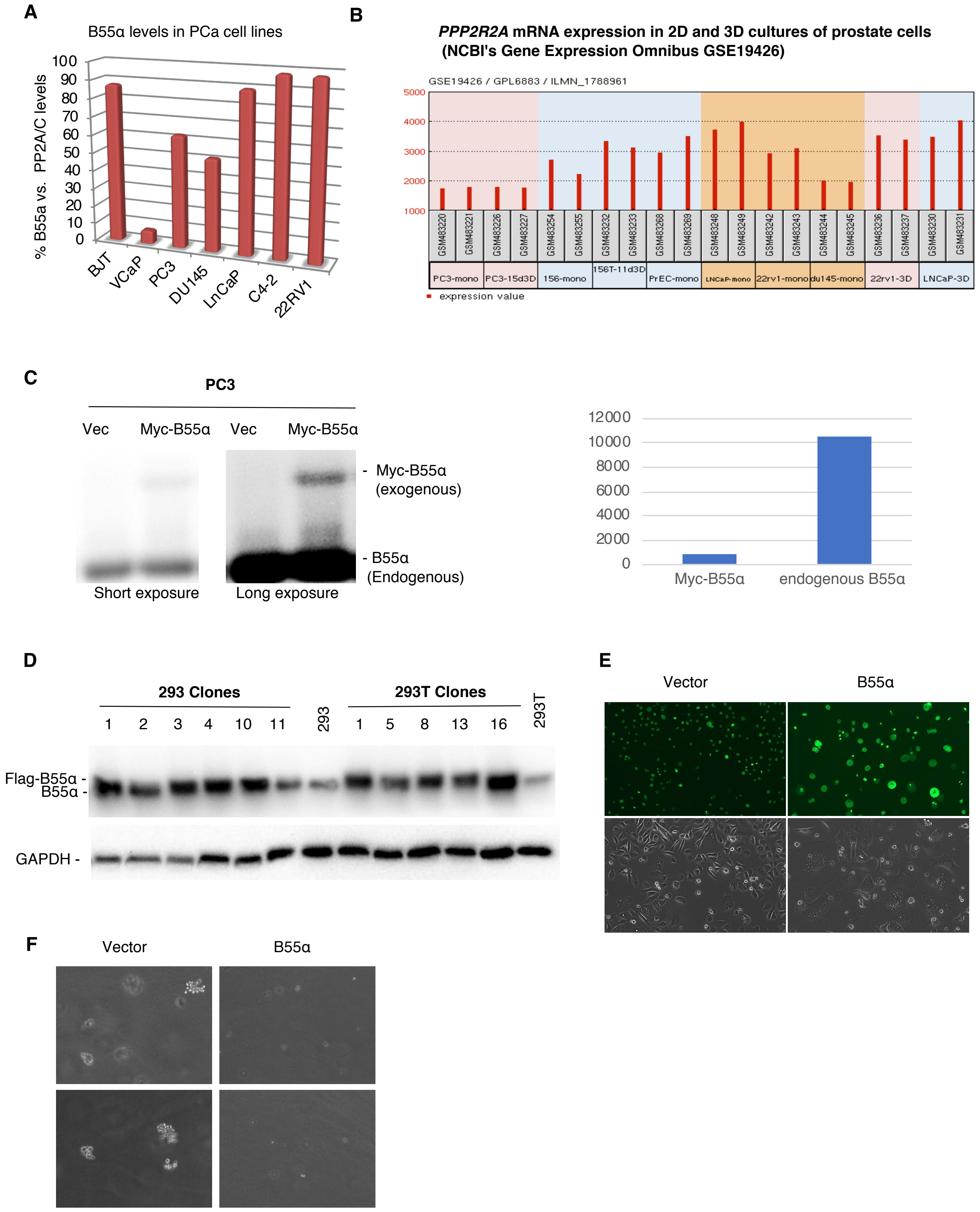

Supplemental Fig. 2.

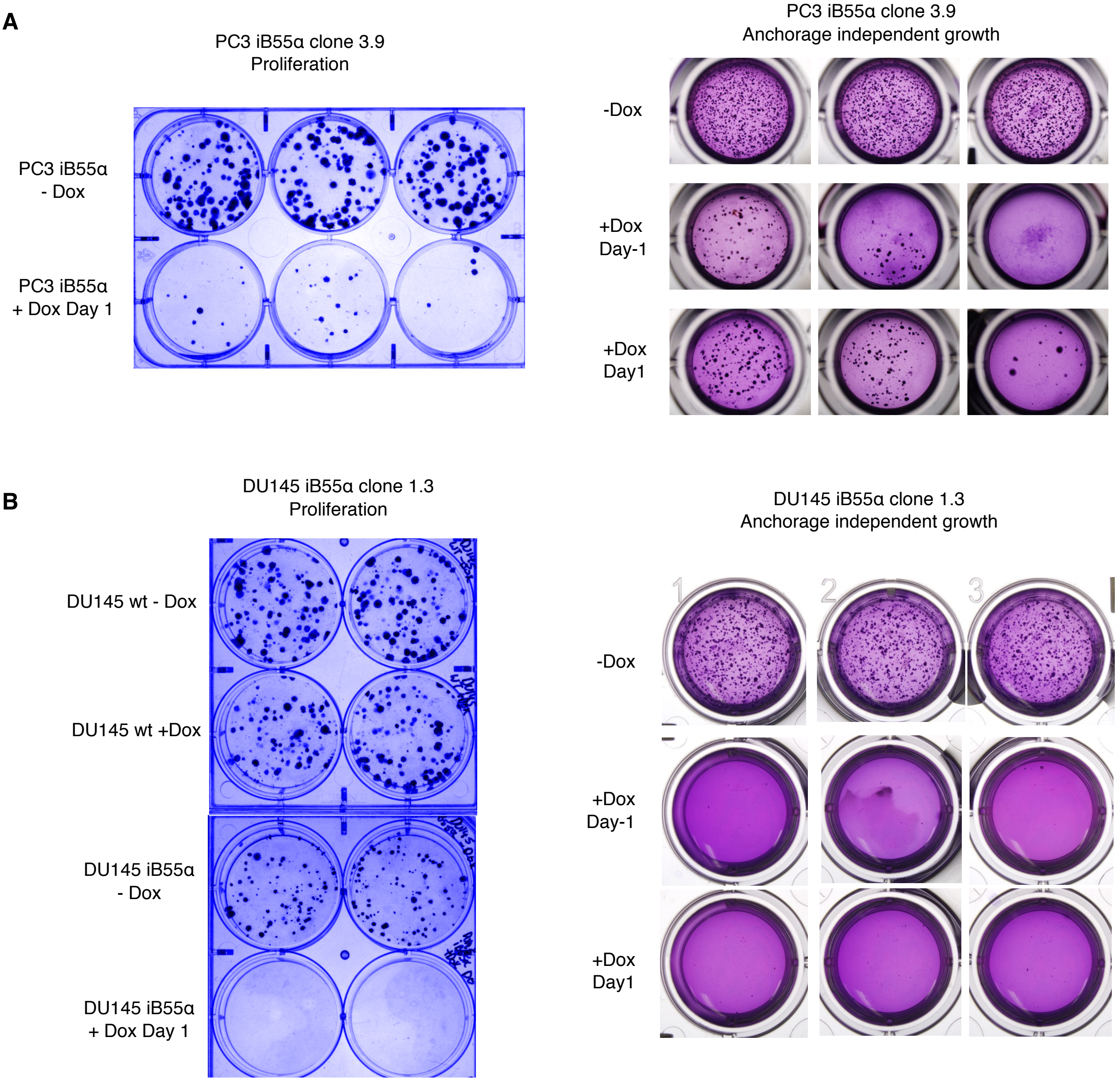

Supplemental Fig. 3.

A

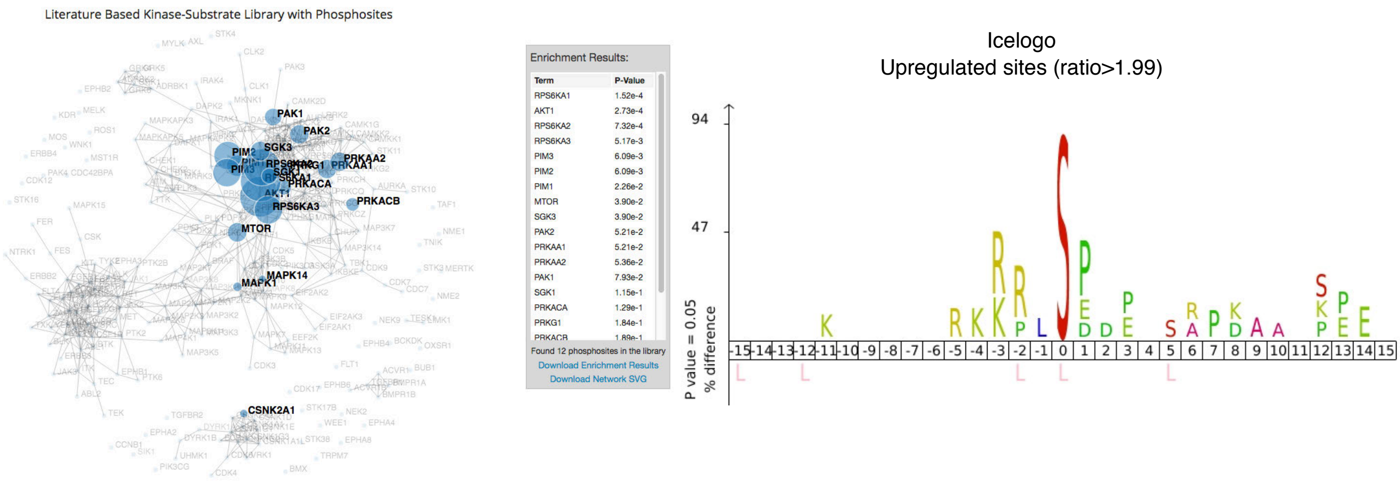

B

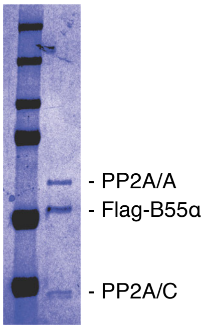

Supplemental Fig. 4.

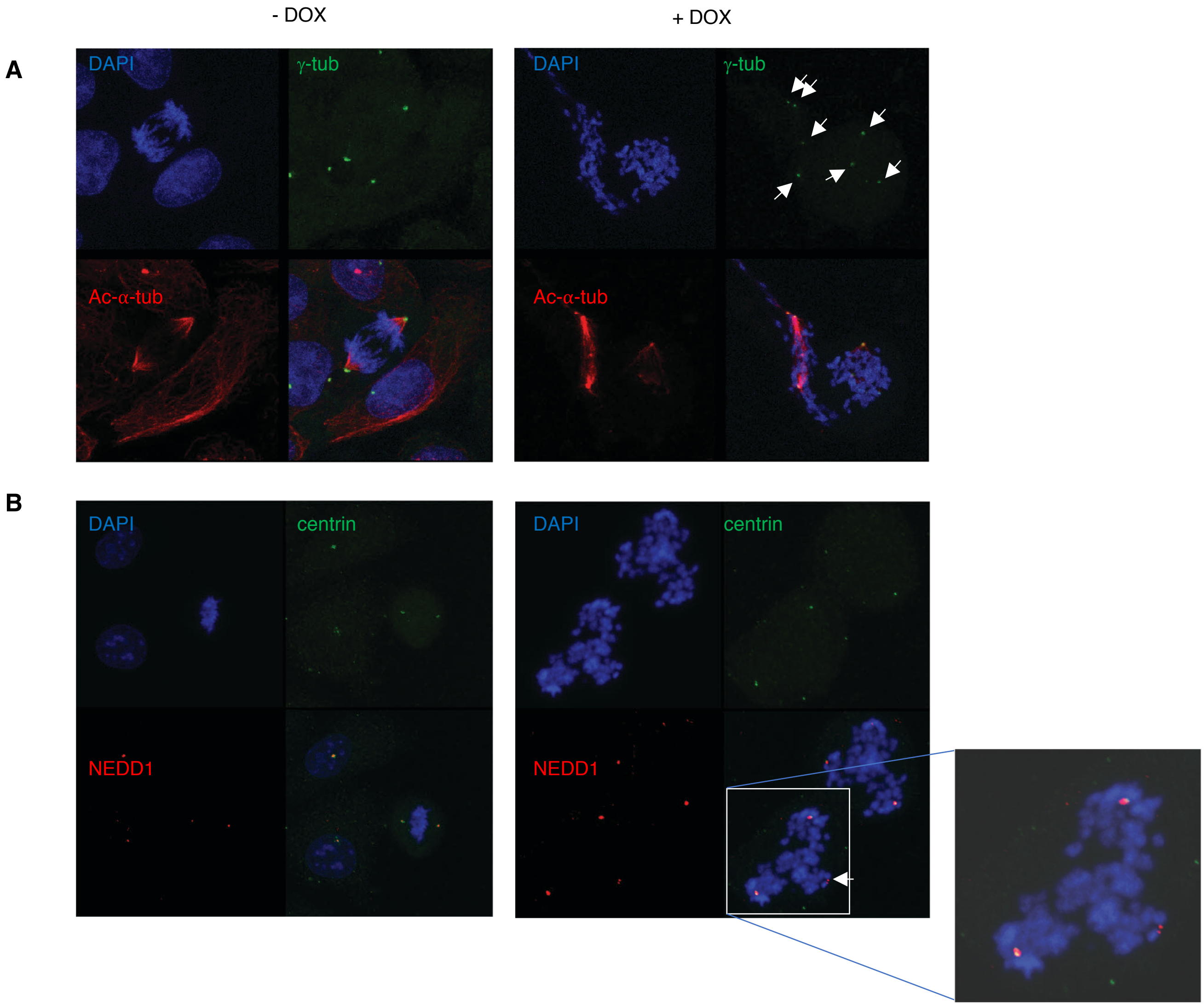

Supplemental Fig. 5.

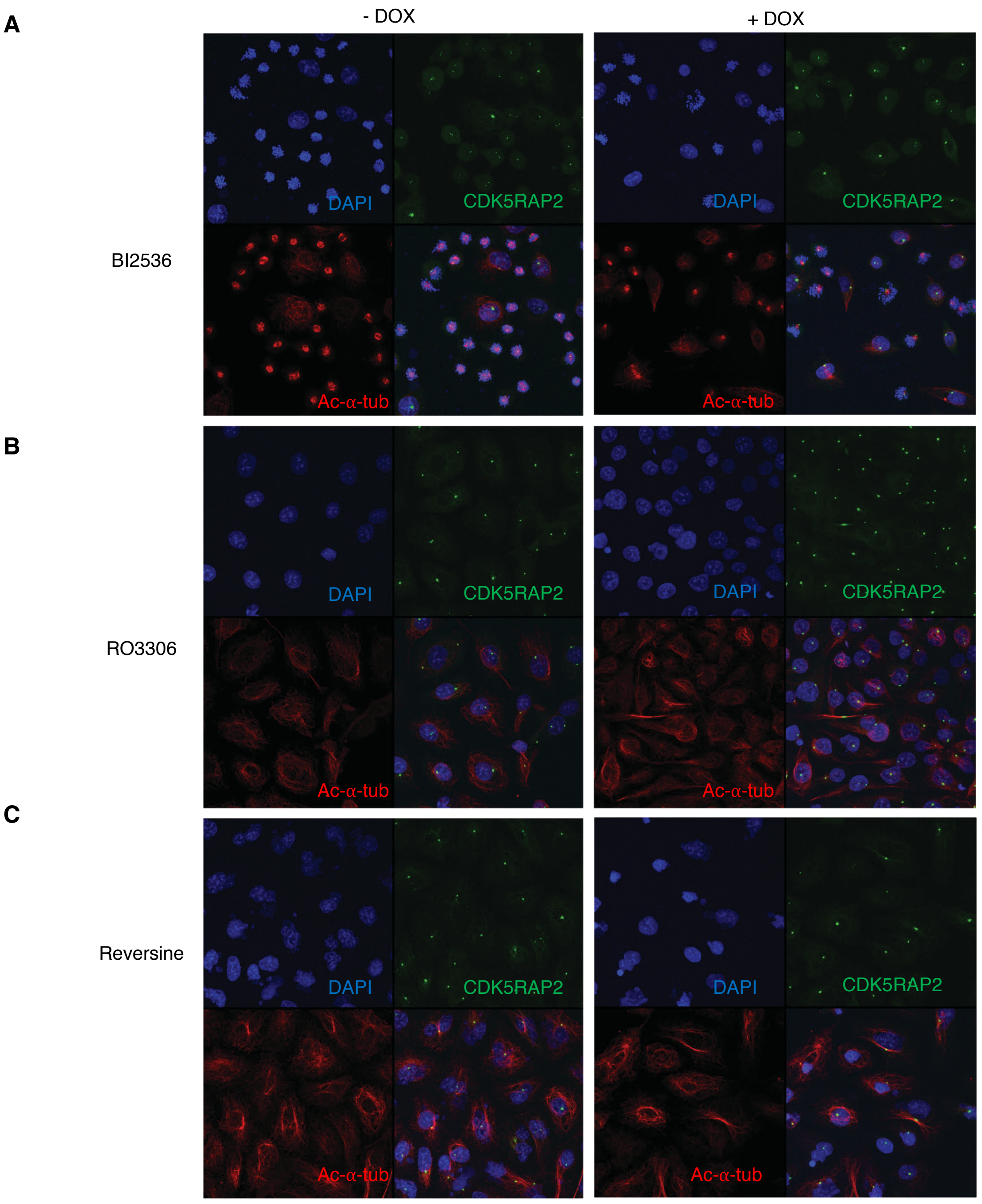

Suppl. Fig. 6.

A

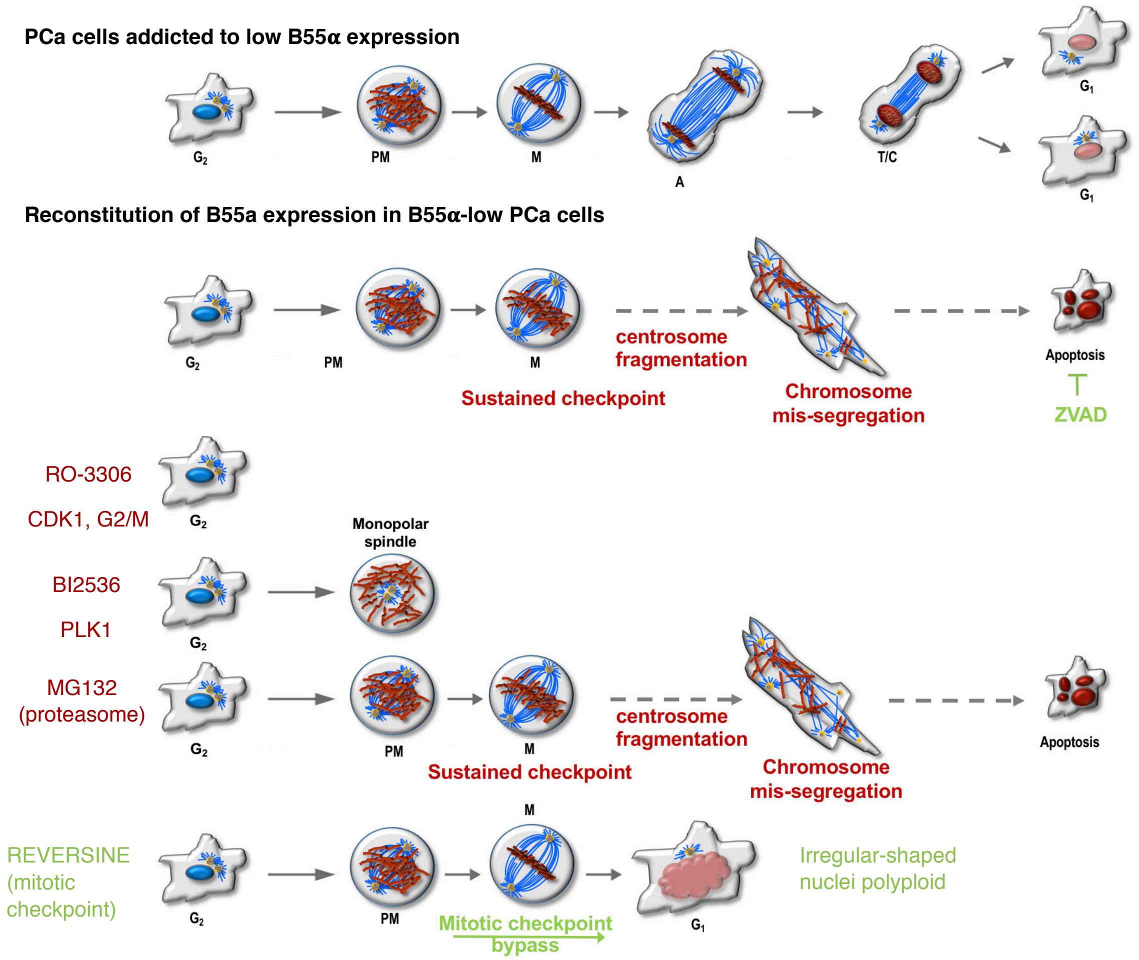

B

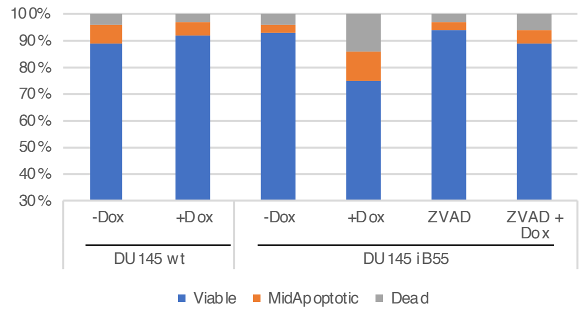

C

+Dox/ZVAD 50 μM

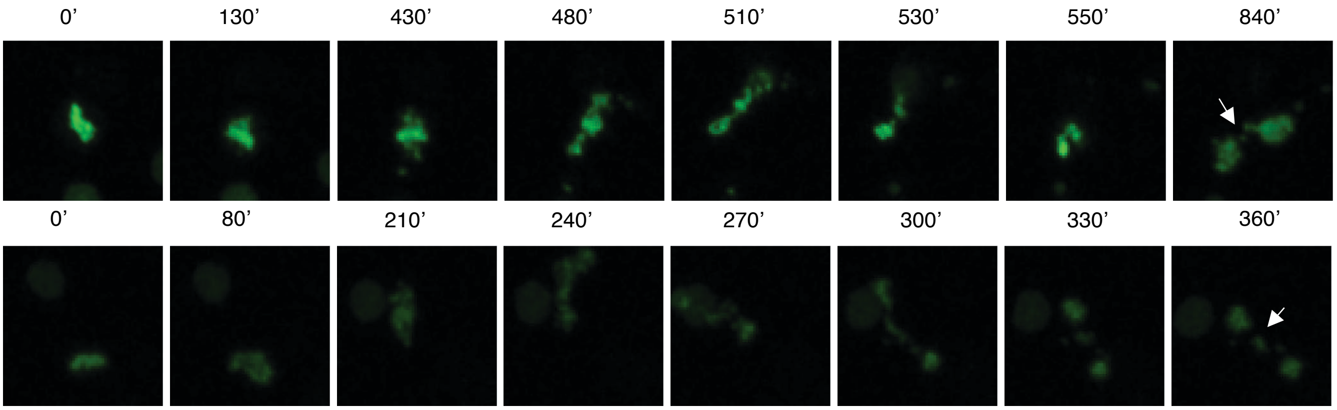

Supplemental Fig. 7.
